# Legal Complexities of Animal Welfare in Australia: Do On-Animal Sensors Offer a Future Option?

**DOI:** 10.3390/ani11010091

**Published:** 2021-01-06

**Authors:** Jaime Manning, Deborah Power, Amy Cosby

**Affiliations:** Institute for Future Farming Systems, School of Health, Medical and Applied Sciences, CQUniversity Australia, Rockhampton, QLD 4701, Australia; d.a.power@cqu.edu.au (D.P.); a.cosby@cqu.edu.au (A.C.)

**Keywords:** animal welfare, legislation, precision livestock farming, livestock, on-animal sensors, five domains, five freedoms

## Abstract

**Simple Summary:**

‘Good animal welfare’ has evolved in recent decades to recognise behavioural, physiological and health factors, acknowledging that an animal may have good clinical health and be productive, though their welfare may be poor. The five freedoms and domains of animal welfare provide internationally recognised frameworks against which to evaluate practices to shape evidence-based standards which recognise both the physical and mental health needs of animals to provide a balanced view of an animal’s ability to cope in its environment. Whilst there are many techniques to measure animal welfare, the challenge lies with how best to align these with future changes in definitions and expectations, advances in science, legislative requirements and technology improvements. Substantial literature discusses the use of technology for improving animal monitoring, management and productivity on and off farm, though little has been published in relation to using such technologies to support legislative compliance and drive overall improvements in animal welfare. This article discusses the current legislation around animal welfare (with a focus on the Australian red meat sector); the impact of public expectation of welfare standards and production practices; and the current and future opportunity for on-animal sensors to support animal welfare, monitoring, management and compliance.

**Abstract:**

The five freedoms and, more recently, the five domains of animal welfare provide internationally recognised frameworks to evaluate animal welfare practices which recognise both the physical and mental wellbeing needs of animals, providing a balanced view of their ability to cope in their environment. Whilst there are many techniques to measure animal welfare, the challenge lies with how best to align these with future changes in definitions and expectations, advances in science, legislative requirements, and technology improvements. Furthermore, enforcement of current animal welfare legislation in relation to livestock in Australia and the reliance on self-audits for accreditation schemes, challenges our ability to objectively measure animal welfare. On-animal sensors have enormous potential to address animal welfare concerns and assist with legislative compliance, through continuous measurement and monitoring of an animal’s behavioural state and location being reflective of their wellbeing. As reliable animal welfare measures evolve and the cost of on-animal sensors reduce, technology adoption will increase as the benefits across the supply chain are realised. Future adoption of on-animal sensors by producers will primarily depend on a value proposition for their business being clear; algorithm development to ensure measures are valid and reliable; increases in producer knowledge, willingness, and trust in data governance; and improvements in data transmission and connectivity.

## 1. Introduction

‘Good animal welfare’ has evolved in recent decades from the focus on subjective measures of wellbeing to recognising behavioural, physiological and health factors [1]. This evolution recognises that an animal may have good clinical health and be productive, though their welfare may be poor [2]. The five freedoms of animal welfare were developed in 1965 by the British Bramble Committee review into the welfare of animals in intensive livestock production systems and later extended by the UK Farm Animal Welfare Council in 1979 to recognise both the physical and mental health needs of animals [1,3]. More recently, the five freedoms have further evolved to the five domains of animal welfare (1994) [nutrition, environment, health, behaviour and mental state] to provide a balanced view of an animal’s ability to cope in its environment [1] and to provide an internationally recognised framework against which to evaluate practices to shape evidence-based welfare standards.

Despite the five domains being internationally recognised, there is a significant disconnect between expectation and practice, with most legislation, standards, guidelines and accreditation schemes in Australia making no specific reference to the domains, or if mentioned, do not provide sufficient detail or guidance on clear compliance requirements and measurement methods. Whilst there are many accepted techniques to measure animal welfare using the five domains, the challenge lies with how best to align with the future changes in definitions and expectations, advances in science, legislative requirements, and technology improvements.

This article will discuss the current legislation around animal welfare related to the Australian red meat sector, and the impact of public knowledge and expectation on welfare standards and production practices. Additionally, the current and future opportunity for technology to support animal welfare monitoring, management and compliance outcomes will be explored. Whilst substantial literature discusses the use of technology for improving animal monitoring, management and productivity on and off farm, little has been published in relation to using such technologies to support legislative compliance and drive overall improvements in animal welfare.

## 2. Animal Welfare Law

The effectiveness of animal welfare legislation depends on the perceived legal status of the animal and the recognition of sentience. However, vagaries in wording and enforceability may be subject to interpretation by industry, courts and juries [2], as described by Wolfenson (2020):

“The ideal welfare law applies to all sentient animals, is clearly written, includes failing to meet an animal’s needs among the offences, is easy to amend in line with new scientific knowledge and ethics, has high legal status which allows for prosecutions, has a clear enforcement responsibility, involves an enforcement body with sufficient power and funds, and includes education of the public and industry”.

[2]

### 2.1. Australian Animal Welfare Law

The objective of legislation in Australia is to prevent animal cruelty, with changes in recent decades reflecting increased public awareness and sentiment for welfare issues [4]. A lack of power under the Australian Constitution means that individual States and Territories are responsible for issuing laws for animal welfare and protection resulting in eight different pieces of legislation aimed at preventing cruelty. The Commonwealth’s involvement in the welfare of farm animals is in the form of Model Codes of Practice (MCoP) or the more recently updated Australian Animal Welfare Standards and Guidelines (AAWSG). The AAWSG for cattle was agreed upon by Australian States and Territories in 2016, replacing the 2004 Model Codes of Practice for the Welfare of Animals—Cattle [5]. The AAWSG is comprised of objectives, standards and guidelines for aspects surrounding the management and husbandry of the respective livestock species. Objectives are the overall intended outcome of the standards and guidelines. Standards are those obligations which should be met by any person in charge of animals to ensure legal requirements are met. Guidelines outline the recommended practices to achieve the desired animal welfare outcomes; often exceed requirements in the standards; are not intended to define best practice; and non-compliance does not constitute an offence. To mandate the compulsory compliance to the terms of the AAWSG, they must be incorporated into animal protection laws of each jurisdiction.

For example, in Queensland, the primary legislation concerned with animal welfare is the *Animal Care and Protection Act 2001* (the Act) [6]. The purpose of the Act (s3) includes to promote the responsible care and use of animals (domestic and production), ensuring that they are protected from unnecessary, unjustifiable and unreasonable pain. It sets out to do this whilst achieving a reasonable balance between the interests of animals and the people who derive a livelihood from them [6], recognising that advances are being made in the knowledge of animal biology and the changing expectations of the community. Section 4 of the Act states that the purpose can be achieved through the requirement to comply with codes of practice as outlined by regulation. Only two national codes of practice are compulsory in their entirety—land transport of livestock [7] and the Australian code for the care and use of animals for scientific purposes [8]. Under the *Animal Care and Protection Regulation 2012* (the Regulation) [9], certain provisions of the MCoP for the welfare of animals—domestic poultry (4th edition) and pigs (3rd edition)—are compulsory. The AAWSG—Cattle is not mentioned in the Regulation. However, compliance with its predecessor, the Australian MCoP for the welfare of animals—Cattle is voluntary. It is reported that compliance with the AAWSG—Cattle will be made compulsory in Queensland under the Act. However, the date of implementation is as yet unknown [5]. Implementation of AAWSG—Sheep in Queensland is also currently outstanding [10].

The enforcement of the Act impacts its effectiveness and the longer-term ability to improve animal welfare in Queensland. Current barriers to enforcement of livestock infringements in particular are geographic distances, reluctance to report issues and confusion by both the public and livestock sector as to the correct reporting agency and process [4]. Responsibility for enforcement is often spread between insufficiently resourced departments with dual priorities of enforcement and promotion of the industry [2,4]. Within each Australian jurisdiction, enforcement of animal welfare law is shared by the relevant RSPCA (Royal Society for the Prevention of Cruelty to Animals) state body and government agencies [4]. The RSPCA are focused on companion animals and government agencies responsible for enforcement of animal laws relevant to livestock. Animal welfare inspectors employed by the enforcement body have the powers of entry, inspection, seizure, animal destruction and to issue orders using the MCoP. In Queensland, Biosecurity Queensland (a service area of the Department of Agriculture and Fisheries) and the RSPCA Queensland work in partnership to monitor compliance and provide public education [11].

Prosecution rates in Australia are similar to other developed countries at less than 1% of case reports, likely due to under-resourced enforcement agencies pursuing action only in cases with a high likelihood of a guilty verdict [4,12]. Furthermore, low prosecution rates are a result of over-reporting due to a lack of public knowledge of actions constituting an offence [4]. Successful prosecution under the Act for the offences of cruelty or failure of duty of care require the criminal standard of a high burden of proof to establish that harm has occurred beyond reasonable doubt [4,12]. Even if determined, orders for prosecution are often overturned on appeal or invalidated by Commonwealth law (in the case of export industries) [12]. Limited resources for investigation, extensive nature of livestock properties and the associated large geographic distances for livestock inspection and confusion over compliance exemptions for adherence with AAWSG standards often prevents litigation being instigated in time, particularly given relatively short statutes of limitation ranging from one to four years across the States. The AAWSG also acknowledge that, in relation to standards, “science cannot always provide an objective or precise assessment of an animal’s welfare and, consequently, where appropriate science is not available, the standards reflect a value judgement that has to be made for some circumstances” [5], further compounding the vagaries of the legislation.

### 2.2. Animal Welfare Law in Other Countries

Compliance and enforcement issues are not unique to Australia. As more countries seek to implement animal welfare legislation and accreditation schemes based on the five domains framework, loosely enforceable standards and guidelines without clearly definable and measurable welfare measures will continue to hamper enforcement efforts. The detail, clarity and extent of animal welfare legislation adopted internationally also varies significantly between developed and developing countries. A thorough review of international animal welfare legislation has not been conducted for this paper. Aspects of the relevant law in the United Kingdom (UK), European Union (EU) and USA have been considered due to those regions being significant influencers in the red meat sector. Norway, Denmark and New Zealand are included due to their history of seeking to achieve best practice in relation to livestock handling. In the UK and EU, compliance and enforcement activities target those businesses posing the greatest risk of non-compliance [13]. Producers seek to join private or accredited schemes to earn ‘points’ to reduce the frequency of government compliance reporting and inspections [13]. Historically in the UK, compliance officers identified more breaches for record keeping than poor animal welfare [13], placing doubt over whether legislation is meeting objectives. Currently in development, the Animal Welfare Assessment Grid (AWAG) intends to provide an evidence-based, technology tool for continual welfare assessment for decision making at the farm level and to aid legislative compliance and accreditation [2,14]. With limited information currently available publicly, no assumption on the likely effectiveness has been made, though presenting a future review opportunity.

The Danish government sets high standards for animal welfare, often exceeding EU directives [15]. The Denmark Animal Welfare Indices (DAWIN) [15] for pigs and cattle were developed in 2016 to provide transparent, valid, practical, evidence-based measures in line with the Welfare Quality [16] definition of welfare focused on the animal’s experience in its environment. The purpose of DAWIN is to allow national surveillance of the livestock industry to inform future change in practice and legislation, though such success will be dependent on the measures being accurate, objectively recorded, consistent and feasible to implement at the farm level [17]. From 2021, Denmark’s animal welfare legislation will recognise all animals to be sentient beings, with specific rules for all species including livestock. Measures and enforcement details have not been reviewed for this article.

Norway sets high animal welfare standards and recognises the significant resource-burden of inspection and enforcement. The current structure and processes were implemented in 2010 and include random and risk-based audits. To attempt to reduce the burden of inspection and audit, initial animal welfare reports/complaints are directed to a community panel to determine if investigation/enforcement is required and by endorsing suitably trained veterinarians as animal welfare inspectors [12].

The USA livestock industry relies largely on market regulation, assuming consumers will incentivise producers through price and demand increases for high animal welfare products [18]. Lack of prescription by USA state and federal authorities makes animal welfare laws largely ineffective, with most regulations focused on the meat processing sector, and robust, independently audited on-farm schemes largely absent [18]. Some headway has been made into the assessment of welfare on farm through the introduction of Global Animal Partnership (GAP) measures in 2008 [19] and the Common Swine Industry Audit (CSIA) in 2014 [20]. The CSIA aims to provide a consistent, reliable, and verifiable system, with detailed assessable criteria verified and numerically scored by third-party audit. Though not subject to a thorough review for this paper, the current shortcomings include there being no minimum passing score; the third-party auditor may or may not have certified credentials (CSIA states ‘ideally certified’); the audit is not compulsory, and; the ultimate determination of sale of livestock rest on the decision of the processor to accept or reject supply based on the audit score [20]. Following initial accreditation, the GAP requires that reaccreditation by an on-farm audit of all properties is conducted every 15 months, with inspections covering all animals on site [21]; demonstrating the highest compliance requirement of the red meat accreditation schemes reviewed for this paper.

Animal welfare legislation in New Zealand is structured similarly to Australia, with the overarching *Animal Welfare Act 1999* (the NZ Act) [22] containing regulations and codes of welfare (both mandatory) along with voluntary recommendations which provide guidance on care and conduct above the minimum requirements set by the regulations and codes. Animal sentience based on the five freedoms was recognised in the NZ Act in 2015 and planned to be incorporated into codes of welfare during future review processes [23,24]. Currently, however, updated codes of welfare for selected industries [Meat Chickens (2018), Sheep and Cattle (2018), Dairy Cattle (2019)] [25] do not yet contain mention of animal sentience or the five freedoms framework, perhaps highlighting the challenge of developing evidence-based measures and benchmarks to determine compliance.

Changes to animal welfare legislation overseas will likely influence government and industry policies in Australia. The Australian Capital Territory is the first jurisdiction in Australia to legally recognise animal sentience (September 2019) [26]. However, this is currently for companion animals only, further highlighting the complexities of developing provisions in legislation for animal sentience in livestock. In a global context, it will be important to consider how current provisions can be extended beyond the mere recognition and acknowledgement of animal sentience, to the development and implementation of robust measures of animal wellbeing which are reliable, accurate, conceivable to measure by producers and, most importantly, be a true reflection of an animal’s experience in its environment.

## 3. Producer Compliance and Consumer Expectation and Knowledge of Animal Welfare Practices

### 3.1. Demonstrating Producer Compliance

Since 2016, through voluntary registration with the Livestock Production Assurance (LPA) scheme, Australian red meat producers must complete an LPA Biosecurity Plan (template provided by LPA) which includes animal welfare requirements to demonstrate that livestock handling is consistent with the requirements of the AAWSG for cattle, sheep and goats. On-farm LPA audits are conducted on approximately 1% of randomly selected producers annually [27]. Audit compliance requires that producers must have a copy of the AAWSG available (or accessible) and maintain records to demonstrate that the producer (and all staff involved in animal husbandry) complete the LPA animal welfare online training module and associated assessment (proof by certificate of completion) [27]. This is intended to demonstrate that livestock handling practices are consistent with AAWSG and enable exemption from prosecution for animal cruelty or breach of duty of care under the Act, despite AAWSG not yet being mandatory in most states.

Greater public scrutiny and higher expectations of improvements in animal welfare are likely to place pressure on the Australian red meat sector to more actively demonstrate compliance with AAWSG in future. Based on experience overseas, it is likely that LPA compliance standards will be required to move beyond the mere acknowledgement of the AAWSG and confirmation of training module completion, to comparison of detailed animal handling practices against known high standards, more in line with international frameworks. To improve animal welfare, future audit and compliance requirements need to ensure that the focus is on producers being encouraged to continue and enhance good animal management practices, rather than being measured on their ability to complete paperwork [13].

### 3.2. Consumer Expectations of High Farm Animal Welfare

The evolution of animal welfare definitions and media attention of poor animal welfare examples has increased public expectations to make improvements and the need for greater transparency of on-farm practices [28,29,30]. The media play a significant role in animal welfare confusion [2,31], shifting consumer preferences and driving organisations to develop and market socially responsible products [32]. Additionally, animal welfare strategies are increasingly included in corporate social responsibility (CSR) policies [33,34], recognising that shareholders and the public expect compliance with community expectations [12,30,32] and practices. Failing to gain a social license may lead to community opposition, activist action, legal or government intervention [35]. This has the potential to drive change in the animal welfare standards of companies across the supply chain as the absence of CSR policies may reduce investment funding opportunities [12,36,37], decrease transparency, increase stakeholder criticism, and tarnish their reputation [30]. Therefore, it is critical that measurement methods of animal welfare continue to evolve to ensure that CSR promises offer tangible benefits without onerous impositions on producers.

### 3.3. Consumer Knowledge of On-Farm Livestock Management Practices

While the public are increasingly concerned about farm animal welfare and the perceived poor standards and lack of transparency in production methods, their actual knowledge of on-farm agricultural practices is low [28,38]. The public source most animal welfare information from the internet, media and friends/close contacts, resulting in opinions based on inaccurate or biased reporting [2,39], focused mostly around the living arrangements of certain animals [38,40]. Their belief is that animals have a better quality of life if raised more naturally [28], driving their demand for more information regarding production methods and welfare [41,42]. Yet, the average consumer does not feel that their individual purchases impact the market and consider that legislation is more influential in driving change [43]. While the public expects improvements in animal welfare, there is limited knowledge by consumers around the law and what constitutes an offence [4]. The public expect the government should exercise power to create reform in animal welfare [40], currently viewing standards as inadequate, resulting in natural suspicion in regards to accreditation programs [39]. This risks the ongoing potential for the media to lead the uninformed population into the animal welfare debate, leading to extreme government decision making [40], as evidenced by the temporary Australian live cattle export ban in 2011.

### 3.4. Consumers’ Willingness to Pay (WTP) for Higher Animal Welfare Products

Most producers employ high levels of animal welfare, demonstrated through record keeping, third-party audits and an overall desire to make improvements. However, this can have significant cost implications for producers, requiring consumers to be willing to pay higher prices for improved animal welfare practices above the bare minimum required by legislation [44,45]. Consumer WTP for higher farm animal welfare products is influenced by socio-economic status, knowledge of animal welfare issues and trust in such claims [33,44,46]. While consumer demand for higher animal welfare products does have the potential to drive up welfare standards [38], this is only one determinant of purchasing behaviour (along with cost [43], quality attributes [42], perception of quality/health benefits [31]). Hence, the higher WTP generally does not translate into actual purchasing behaviour (the welfare–preference paradox) [18,31,43]. This conflict between ethical consumption and spending [43] is evidenced by higher demand at the lower price point, leading to an increase in intensive farming practices (particularly in poultry and pig industries) at the risk of potentially lower animal welfare [18].

## 4. Welfare Assurance Schemes

### 4.1. Animal Welfare Assurance Schemes

Societal concern has influenced the development and uptake of assurance schemes which recognise improved stewardship in animal welfare [29,45]. Producers are motivated to participate in voluntary assurance schemes to gain consumer/stakeholder appreciation, which may differentiate their produce in the market, increase price and maintain/open access to markets [34]; however, adoption by producers is dependent on the labour, time and cost impositions [41,45].

As evidenced by acknowledged limitations of current animal law in Australia, much of the detail of how best to provide and measure high animal welfare in the livestock industry has shifted to a more voluntary framework. In the absence of prescriptive legislation, the adoption of voluntary assurance schemes enables producers to demonstrate higher on-farm welfare methods and enable marketing and labelling of their farm and products with positive claims.

The meat product purchasing policies of major supermarket chains and the fast-food industry are well publicised in store and in the Australian media, influencing public awareness in relation to animal welfare issues. Major Australian supermarkets sell more than 70% of the domestic consumption of fresh meat [47] and actively market the availability of higher welfare products including stall-free pork, cage-free eggs, RSPCA-approved chicken and hormone-free beef [28]. Public awareness of their higher welfare products is aided by in-store, online and media advertising and celebrity chefs to endorse the use of such products. Their responsible sourcing policies (part of their CSR policies) state their efforts to support animal welfare in line with the five freedoms [48,49], although supply chain information, accreditation and compliance information is not readily available.

### 4.2. Examples of Animal Welfare Accreditation Schemes in Australia

#### 4.2.1. RSPCA Approved Farming Scheme (AFS)

Commenced in 1996 and reviewed every five years, the RSPCA AFS grants licenses to use the RSPCA AFS logo on eggs, pork, chicken and turkeys, farmed salmon and dairy veal. RSPCA inspectors audit licensees at least twice annually against standards which exceed the MCoP, aimed at improving animal welfare to meet the five domains. Logo use on products leads to increased public awareness, with the assumption of this increasing demand for higher animal welfare products [50]. It should be noted, however, that the RSPCA impact reports on the AFS (2016 [51], 2018 [50]) make no mention of the five freedoms or five domains.

#### 4.2.2. Livestock Protection Assurance (LPA)

As previously mentioned, LPA is the voluntary, industry-managed, on-farm assurance scheme for Australian red meat since 2016. Animal Welfare is one of the seven elements (added in 2017) of the Biosecurity Plan producers are assessed on [27], with accreditation loosely based on AAWSG. Initial accreditation for primary producers was automatic in 2016, placing no onus on the producer to focus on understanding the requirements and self-auditing their operations. Annually less than 1% of producers are audited, with audits conducted by AUS-MEAT (an industry not-for-profit organisation offering training, certification and auditing) and taking 2–4 h per property. Audits cover all seven elements of the LPA, limiting time focused on animal welfare criteria. A producer having the standards available and showing evidence of completion of the LPA training module is little guarantee that on-farm practices are in line with standards.

#### 4.2.3. Pasture-Fed Cattle Assurance System (PCAS)

PCAS is a voluntary, industry-run assurance scheme which enables producers to substantiate claims of pasture-fed production, low-stress handling pre-slaughter, grain/hormone-free status and lifetime traceability. Producers register based on self-audit, followed by a third-party on-farm audit at the producer’s expense [52]. Membership has been impacted due to the cost of compliance being higher than PCAS-alternative schemes offered by Australia’s largest beef processors (Teys Australia and JBS).

#### 4.2.4. Teys Grassland Pasture Fed Standard

A PCAS-alternative accreditation scheme for suppliers of beef to Teys (Australia’s second largest beef processor [53]), incorporating traceability, lifetime grass fed and grain/hormone/antibiotic free is Teys Grassland Pasture Fed. If producers are not currently PCAS-accredited they may receive accreditation in the Teys scheme via initial self-audit, followed by a Teys desktop audit. At Teys’ expense, annually 15–25% of producers are selected for an on-farm audit by inspectors who may include cattle buyers trained in inspection requirements [54]. As Teys pays for the audits, the scheme offers lower cost accreditation for producers than PCAS.

#### 4.2.5. Global Animal Partnership (GAP)

Established in 2008 in the USA, GAP is a five-tiered livestock accreditation system, with higher tiers demonstrating higher animal welfare standards including access to an enriched environment, outdoor access, supplementary feeding and time on transport [19]. Livestock Integrity Systems Australia were accredited by GAP in 2019 as certifiers in Australia and New Zealand. Following initial producer registration, an on-farm audit is conducted to determine the GAP animal welfare rating (and tier), with audits conducted of all accredited farms every 15 months [21,55]. At audit, continued accreditation requires all animals on farm to be inspected and meet the appropriate rating [21].

Assurance schemes tend to reflect what the public desire for livestock species (methods mostly focused on living conditions) to help mitigate risk of public outrage [23]. The common theme across Australian accreditation schemes is the reliance on self-audits, infrequency of random audits, real or perceived lack of independence of third-party or industry auditors and the lack of benchmarking against robust measures [23].

### 4.3. Consumer Certainty about Assurance and Certification Schemes and Product Claims

Used either in conjunction with accreditation schemes or independently, product labelling provides the opportunity for products of high animal welfare to be differentiated in the market. Labelling with images or statements suggestive of more ‘natural’ or ‘animal friendly’ production methods increases product attraction [31]. However, most labels omit the specifics of animal welfare methods, with details not readily available to consumers [43]. In Australia, standards for credence claims are lacking [31,34], with most labelling being voluntary, self-audited and containing vague terms and/or images which may not reflect actual production practices. Legal recourse against credence claims in relation to practices or products need to demonstrate the claims to be sufficiently misleading or deceptive to be captured by Australian Consumer Law [56].

## 5. How Can on-Animal Sensors Be Used to Support Animal Welfare Outcomes in the Current Legal Framework?

Assessment of animal welfare is critical for ensuring compliance with legislative requirements and for improving quality of life. Current compliance audits provide a snapshot of animal welfare mostly at a single time point and do not reflect cumulative stresses [2]. Record keeping is critical to meet current and future animal welfare legislative and accreditation requirements. As demand for more detailed information increases, so too will the compliance burden for producers if they continue to rely on manual recording methods. Tightening of enforcement action against these measures will need to be met with improved data systems and methods for producers to gather, interpret and make informed decisions. Failure to not do so will result in overburdening producers, risk the quality of output information, and make little impact on the overall objective of improving animal welfare. Intensification of production to meet future food security and increasing consumer demand will further expand the need to objectively measure animal welfare. Whilst further development and uptake of technology to monitor behaviours has the potential to lead to significantly less human–animal interaction [57], favourable animal welfare outcomes are possible despite intensification.

Precision livestock farming (PLF) technologies have the potential to record and measure animal behaviours and in turn their welfare state in greater detail than what can be recorded by producers [57]. An additional benefit is autonomous and continuous monitoring in real time, that is non-intrusive, thereby allowing the collection of information without stressing or interfering with the normal behaviour of animals [58,59]. This also allows monitoring in locations where human observation is unavailable or difficult [60]. Furthermore, animal welfare has the potential to be increased when monitoring livestock at an individual, as opposed to herd level [29]. While the AAWSG do not specifically mention the five domains, future use of PLF by producers needs to ensure that the systems are capable of tracking, monitoring and reporting activities which support and improve the animal’s wellbeing in line with these.

Current PLF technologies (and those in development) include those worn on animal (RFID, on-animal sensors, GPS collars, biometric sensors, implantables) or off animal (drones, cameras, walk over weigh scales). RFID devices (either in the form of ear tags or collars) allow animals to be individually identified and for information to be linked to other technologies [35], allowing collection and utilisation of data to aid both production and welfare requirements. The use of RFID technologies on livestock has increased significantly in the past decade, with RFID ear tags now mandatory for cattle in Australia, sheep in Victoria (a state of Australia) and voluntary for sheep and goats in other Australian jurisdictions. This movement towards more widespread adoption will pave the way for future collection and reporting on production and welfare measures beyond those currently available. As technology and the development of reliable measures of animal welfare advance, producers may be encouraged to engage with more complex technologies [35], e.g., on-animal sensors that provide more than just individual identification of livestock. Due to the enormous potential of on-animal sensors to address animal welfare outcomes and compliance, they will be of focus of this paper.

Future advances in PLF are focused on “known location and behaviour” sensor technology (primarily known as ‘smart tags’ or on-animal sensors) to record patterns associated with changes in animal behaviour and activity. In addition to production benefits, these have the potential to provide significant information to support practices which are conducive with high animal welfare and allow an early alert for potential wellbeing issues [35]. On-animal sensors may be placed on all or sentinel animals (a small proportion of livestock in the herd/flock) to reduce cost (at the expense of detail) [58], enabling real-time management and monitoring of individual animals across the red meat supply chain [61] and facilitating autonomous data collection [62]. The potential of unbiased, rapid and more frequent monitoring opens the possibility of future benchmarking of data from producers to allow targeted auditing and compliance assessment of producers with demonstrated results outside of an agreed upon range [23]. At a producer level, future on-animal sensors may be able to provide alerts when the animal’s behaviour and welfare is compromised, ensuring intervention and treatment is not delayed.

On-animal sensors can provide information well beyond manual record keeping and have the potential to address the five domains and animal welfare law:

1. Nutrition
  - Water deprivation—Access to and movement around water points [35] to demonstrate frequency of movement to water.
  - Food deprivation—Access to pasture, location and paddock utilisation patterns [63], either used alone or in conjunction with satellite imagery.
2. Environment
  - Access to shelter [64] to verify provision of shelter.
  - Movement in response to climatic changes [65].
3. Health
  - Movement patterns to alert to health and disease issues such as lameness [66].
  - Predation—herd movement may indicate a predation event, enabling producer intervention [67].
4. Behaviour
  - Use of on-animal sensors for behaviour algorithms [68,69,70].
5. Mental domain
  - Quantification of drinking behaviour to indicate thirst and satiety [71].
  - Socialisation and social interactions [72].
  - Freedom from pain [73].

Additionally, on-animal sensors could enhance livestock production practices and be used to quantify that the five freedoms have been met to improve animal welfare [58].

Future systems need to ensure that producers can accurately interpret the information from the technology [2,58] to support welfare assessments and to allow the information to be used for continuous improvement. Producers are currently unlikely to adopt technology (or expand their use of technology) to allow reporting against guidelines (before they are enforceable) as reporting against measures may highlight issues with on-farm practices, making producers more visible and open to penalties or issues with stakeholders [29]. Producers need to be assured that any technology they adopt fully enables them to demonstrate and measure their current positive practices and is not merely a tool to identify breaches. Conversely, if the technology fails, it does not have an impact on animal welfare [58]. If producers move towards increased monitoring and measurement, consumers and stakeholders also need to be educated about the measures, methods of collection and what it means when deviations are alerted. Over scrutiny of each alert needs to be avoided and kept in perspective so as not to identify and vilify the producer as underperforming on animal welfare measures, sometimes due to circumstances outside of their control.

## 6. Challenges to Adopting On-Animal Sensor Technology

While significant research is being undertaken in Australia and internationally to develop commercially suitable, cost-effective on-animal sensor solutions [35] to improve production, increase efficiency and objectively measure animal welfare state, significant challenges to adoption by producers exist.

### 6.1. Lack of Commercial Options

Ongoing public and private investment to support collaboration between the livestock sector, technology companies, government and research agencies to maximise opportunities is crucial [35,41]. Most existing on-animal sensor technology is stand alone, not well integrated, only used in one aspect of the supply chain [60] and not subject to rigorous trials under commercial conditions to demonstrate success, robustness and suitability [59].

### 6.2. Algorithm Development

While current on-animal sensor technologies have been proven to measure aspects of health, growth, feeding, activity and location of animals, the challenge is how to interpret behavioural responses which may be informative in relation to animal production and welfare [63]. In addition to developing meaningful measures of psychological wellness in line with the five freedoms/domains of animal welfare [60], recognising and evaluating both the positive and negative states is required [2]. Significant further research is required to provide clarity of what constitutes good physical and mental wellbeing for animals before it can be used as a measure, and to acknowledge that animal perception of their environment is different to human perception [57]. Collaborations between engineers, animal scientists and producers are required to develop algorithms for on-animal sensors that address current issues faced on farm and that can accurately determine the behavioural and welfare state of an animal such as confirmation of low-stress handling practices [61]. Algorithms must also be capable of working on different livestock species, property sizes, landscape, and environmental conditions. Finally, producers must be given the opportunity to dispute any claims of poor animal welfare from on-animal sensors, due to acknowledged on-farm challenges such as drought and bushfires which occur. A significant challenge lies in development of these welfare indicators and agreement by the industry and producers regarding what is an acceptable response/intervention to an alert, before these on-animal sensors can be confidently adopted and lead to improved animal welfare [59,60].

### 6.3. Producer Knowledge

Producers are generally unaware of available technology options [74] and are heavily influenced by adoption and outcomes on neighbouring properties [75]. Marketing of current and future technologies needs to reinforce that the data derived aids decision making and intends to support and enhance current practices, cognisant that a producer’s knowledge and use of low-stress stock handling practices cannot be replaced [41,76].

### 6.4. Producer Willingness

Innovation and development of on-animal sensor technologies is no guarantee of producer adoption [75]. Producers require a clear value proposition which offers tangible benefits and the ability to integrate with existing systems and processes [41,62]. Additionally, ease of data collection is important [41], whilst ensuring the information is reliable and valid to avoid rejection of the technology [59,60]. Evidence suggests that technology adopters see the opportunity for profitability [41] and have higher digital literacy skills, with non-adopters feeling the pressure to adopt, having lower digital literacy skills and less funds to spend on technology [76,77].

High upfront capital and installation costs and ongoing updates, servicing and maintenance costs reduce adoption of PLF technologies as return on investment to the producer cannot be clearly ascertained [59,77]. Producers risk software and system compatibility issues with current or future technology choices [77], increasing the potential cost burden. This highlights a need for research to better understand capital and ongoing costs, ease of integration and return on investment of the technology [74]. Further on-farm government or industry funded research, collaborating with small to large producers would help alleviate cost burden to the producer and incentivise uptake [35,62,74]. Potential ownership models of PLF technologies using a ‘service provider’ model whereby the service provider owns, installs, updates and runs the multi-sensors for producers for a fixed annual fee could be considered [78], particularly if future PLF use requires that sensors and data are expected to be utilised beyond the farm gate and along the value chain. Producers will require assurance of training or access to the skills to maintain and repair systems to avoid business interruption [41]; a critical requirement for those in remote locations.

### 6.5. Data Privacy Concerns

Integration of systems to meet the needs of industry along the value chain will require wider collaboration and sharing of data, making the challenge of data ownership critical [60]. Data privacy concerns currently limit producer adoption of existing technologies [77,78], highlighting the need for clear understanding of the use, storage and ownership of data [41,79]. Additionally, there are growing concerns over the misinterpretation of data and the detrimental impacts this information could have for the livestock industry if released to the public [23]. The Australian National Farmers’ Federation voluntary Farm Data Code 2020 follows similar recent developments in USA, UK and NZ to acknowledge significant mistrust by producers in data security and to provide an initial framework for the collection, use and reuse of farm data by service providers [79]. In the future, open-source software development may increase research collaboration and speed development of suitable farm-ready technologies [35]. However, digital governance is critical [41] and will require encryption and segregation of data between users along the supply chain [29].

### 6.6. Data Transmission and Internet Connectivity

Suboptimal internet connectivity in many of Australia’s regional and remote areas is currently a major hurdle for adoption of technologies on farm [41,62,74]. While existing systems are addressing the energy requirements associated for continuous monitoring by on-animal sensors, future research is needed to overcome the challenges of data transmission in low connectivity areas.

## 7. Conclusions

On-animal sensors have the potential to provide significant information to support compliance with current legislation in the Australian red meat industry, though the complexities of determining appropriate measures in relation to animal welfare need significant ongoing research. In the shorter term, adoption of technology to support animal welfare claims will more likely be supported by industry or private company-led accreditation schemes to enable differentiation of premium products. As reliable measures evolve and costs reduce, technology adoption will increase as the benefits across the supply chain are realised. Corporate social responsibility will continue to place pressure on animal welfare improvements at all levels of the value chain, making reliable and easily understood measures critical to support an ongoing social license [35] and becoming the catalyst for greater legislative compliance and enforceability of mandatory standards and guidelines. Legislators are unlikely to mandate the use of technology to support animal welfare [78], as to do so would place increased burden on producers with a larger political voice than ethical consumers [43]. It is critical, therefore, that technology improvements continue to receive industry support to enable reliable reporting of data for compliance and future legislative review and decision making.

## Data Availability

Not applicable.
